# Assessment of Mosquito Collection Methods for Dengue Surveillance

**DOI:** 10.3389/fmed.2021.685926

**Published:** 2021-06-08

**Authors:** Triwibowo Ambar Garjito, Lulus Susanti, Mujiyono Mujiyono, Mega Tyas Prihatin, Dwi Susilo, Sidiq Setyo Nugroho, Mujiyanto Mujiyanto, Raden Ajeng Wigati, Tri Baskoro Tunggul Satoto, Sylvie Manguin, Laurent Gavotte, Roger Frutos

**Affiliations:** ^1^Institute for Vector and Reservoir Control Research and Development, National Institute of Health Research and Development, The Ministry of Health of Indonesia, Salatiga, Indonesia; ^2^Department of Parasitology, Faculty of Medicine, Public Health and Nursing, Gadjah Mada University, Yogyakarta, Indonesia; ^3^HSM, Univ. Montpellier, CNRS, IRD, Montpellier, France; ^4^Espace-Dev, University of Montpellier, Montpellier, France; ^5^Cirad, UMR 17, Intertryp, Montpellier, France

**Keywords:** *Aedes aegypti*, *Aedes albopictus*, dengue, collection methods, dengue incidence, Indonesia

## Abstract

Several methods exist to collect and assess the abundance of dengue vector mosquitoes, i.e., morning adult collection, pupal collection, ovitraps, human landing, and larval collection. Several of these methods are officially implemented to monitor mosquito density and make decisions on treatments for dengue control. This monitoring is also constrained by the need to conduct this assessment on a “one point/one day” process, meaning that once the threshold of 100 households is reached, the assessment is made, and the collectors teams move to another place, thus preventing the use of long-term sampling methods. This diversity of methods might be a source of variability and lack of statistical significance. There is also a lack of published data regarding the efficacy of these methods. Furthermore, the *Stegomyia* indices are shown to be not reliable for assessing the risk of dengue outbreaks. A mosquito survey was, thus, conducted in 39 locations corresponding to 15 dengue endemic provinces in Indonesia by using the different adult and larval collection methods recommended nationwide. A total of 44,675 mosquitoes were collected. The single larva method was the most efficient. Out of a total of 89 dengue-positive pools, the most frequently encountered virus was DENV2, which made up half of the positive samples, followed by DENV3 and DENV1, respectively. Factor analysis of mixed data showed that no correlation could be found between any methods and the presence of dengue virus in mosquitoes. Moreover, no correlation could be found between any methods and the incidence of dengue. There was no consistency in the efficacy of a given method from one site to another. There was no correlation between any of the parameters considered, i.e., method, incidence of dengue, location, and the presence of dengue virus in mosquitoes.

## Introduction

Dengue is the most rapidly spreading arboviral disease worldwide (1). Recent studies estimate that 55–100 million dengue cases are reported annually with 3.9 billion people at risk (2, 3). Indonesia is an hyperendemic dengue country; i.e., all four serotypes are circulating with the highest number of dengue cases in Southeast Asia (4, 5). Dengue incidence in Indonesia has increased significantly over the last four decades from 0.05/100,000 in 1968 to 78.8/100,000 in 2016 (6). The dengue virus is transmitted to humans by the bite of infected *Aedes aegypti* mosquitoes, the main vector, and *Ae. albopictus*, the secondary vector. These species are anthropophilic; i.e., they live in human environments and breed in various sites, such as water containers, flowerpots, birdbaths, disposed water-holding vessels, waste disposal areas, small containers, discarded tires, natural holes in vegetation, etc. (7–10). Both are present in urban and suburban areas. With no treatment and while an effective vaccine is still under study, vector control remains the only effective way to prevent and control dengue.

Vector surveillance methods have remained mostly unchanged for more than three decades (11). Larval survey is the most widely adopted dengue vector surveillance method to locate larval habitats and to measure the abundance of *Ae. aegypti* and *Ae. albopictus* (12, 13). The *Stegomyia* indices, i.e., house index (HI), container index (CI), and Breteau index (BI), to which a specific free larval index (FLI) is added in Indonesia, are used for calculating mosquito abundance and for predicting the risk of dengue transmission (11). The FLI is defined as the number of houses without larva x 100/total number houses. The FLI is, thus, the reverse of the HI. However, previous studies demonstrated the lack of correlation between *Stegomyia* indices and the risk of dengue outbreak (12, 14–18), although a correlation was found between human population density and incidence of dengue (18). This sounds logical owing to the anthropophilic behavior of these vectoring mosquitoes. Several methods for collecting mosquitoes are officially recommended by the Ministry of Health, i.e., morning adult collection, pupal collection, ovitraps, whole night collection using human landing, and larval collection. Because the *Stegomyia* indices, exclusively based on larval collection, are not reliable predictors, other predictors must be sought. The diversity of methods endorsed by the Ministry might be a source of variability. Furthermore, there is a lack of published data regarding the effectiveness of these methods (12, 19–22). A major constraint associated with the monitoring of mosquitoes is that the decision for treatment or absence of treatment is made on a one point/one day basis, meaning that agents in charge of the survey issue the assessment conclusion once the threshold of 100 households is reached and move to another place. They do not conduct long-term capture and sampling. Methods of insect collection influence the reliability of entomological indices. We report here a large-scale comparative analysis of various methods of insect collection to assess their relative effectiveness and reliability to determine whether entomological descriptors can be envisioned to determine the risk of dengue outbreak or if other kinds of descriptors should be considered.

## Methods

### Study Sites

**Figure 1:**
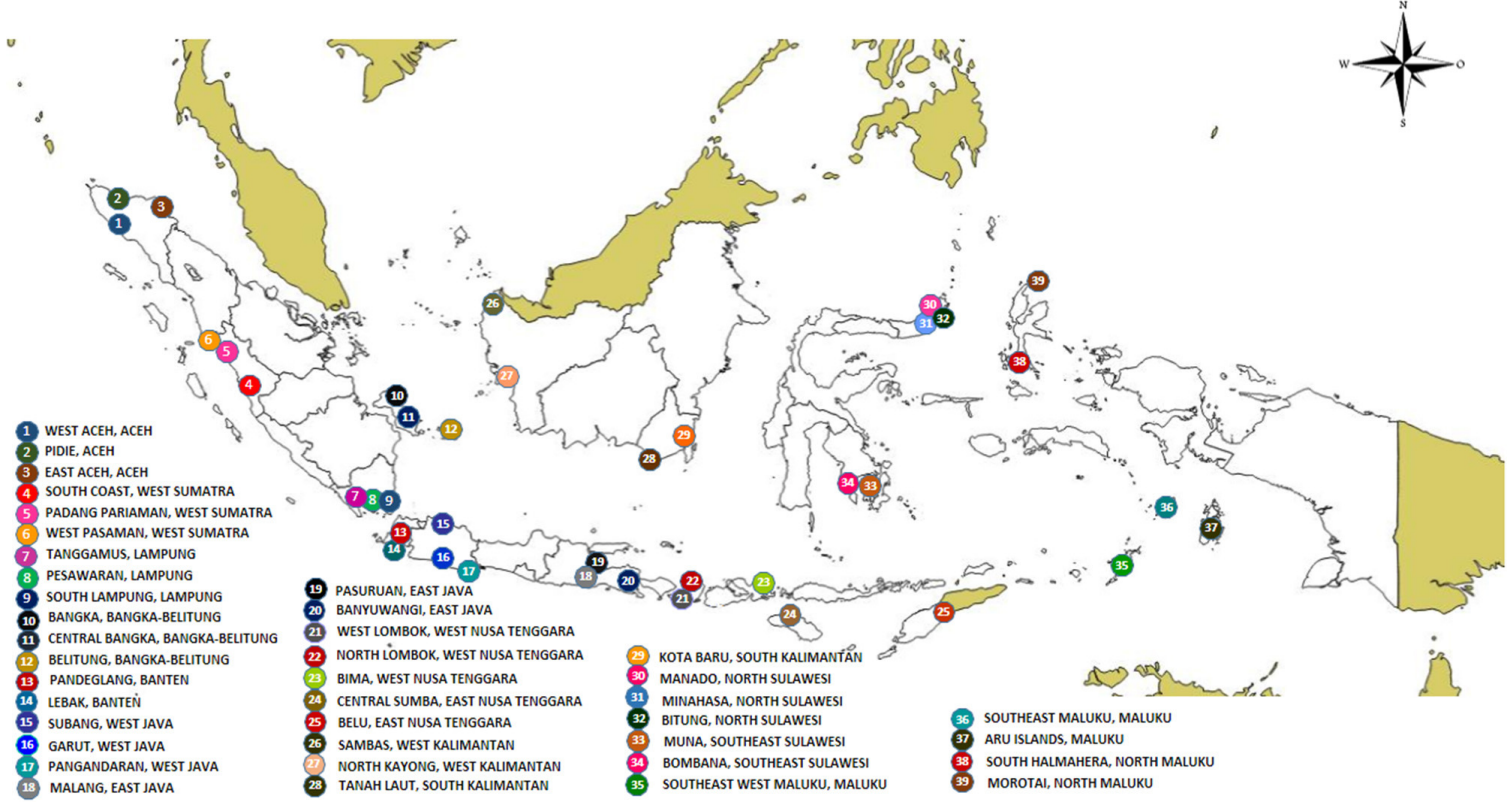
Map of the sampling sites throughout Indonesia.

The study was conducted in 39 locations corresponding to 39 districts/municipalities in 15 dengue endemic provinces in Indonesia (Figure 1). These provinces include Aceh, West Sumatra, Lampung, Bangka-Belitung, Banten, West Kalimantan, South Kalimantan, North Sulawesi, West Java, East Java, Southeast Sulawesi, Maluku, West Nusa Tenggara, East Nusa Tenggara, and North Maluku. This study is part of the Indonesia national project, Rikhus Vektora led by the Ministry of Health which started in 2016.

### Study Design

A mosquito survey was performed in all study sites from July to August 2016 during the rainy season. Larva collection was performed in at least 100 households taken at random in each study site according to the recommendations for the calculation of the *Stegomyia* indices. Adult collection of *Aedes* mosquitoes were performed in the morning (morning resting) on mosquitoes resting inside houses using manual aspirators. Adult mosquitoes were also collected outside using standard procedures for all night human-landing collection methods from 6 p.m. to 6 a.m. All methodologies used in this study are described in the Ministry of Health guidelines (22). All methodologies investigated must be compatible with the “one point/one day” process of decision making. This “one point/one day” concept means that collectors do not stay in the same sampling site over a long period of time. As soon as the threshold of 100 households is reached, they calculate the *Stegomyia* indices and move to another sampling site. Long-term sampling methods are not suitable. Field data collections for larva and adult *Aedes* mosquitoes were performed by trained collectors in collaboration with local volunteers, local authorities, and staff from district/municipality dengue control programs.

#### Single Larva and Rearing Methods

At least 100 households were taken at random in each study site for larval and pupal sampling. All artificial and natural water containers in and around each household were inspected for mosquito larvae and pupae. At least one larva (second, third, and/or fourth instars) and pupae (if any) from each positive container were collected with pipettes and tea strainers. Water from large containers was first removed with a water hose and then sieved with tea strainers. Larvae or pupae were then collected on a white plastic tray. Immature stages were pipetted and placed in a plastic clip with water, labeled, and taken to the field laboratory. Data on infected containers and households were used for calculating the *Stegomyia* indices. This corresponds to the single larva method routinely implemented for mosquito monitoring. For the rearing method, immature mosquitoes (pupae and larvae) were reared in 250-ml plastic cups covered with gauze under room temperature. Larvae were fed on tetrabit fish food until adult mosquitoes emerged. Emerged adult mosquitoes from the same household and species were then killed in a freezer (−20°C) or by using ethyl acetate for 5–10 min and immediately stored in 1.5-ml vial tubes with RNALater (Qiagen, Hilden, Germany) by pools of up to 25 specimens. Mosquitoes from different households or different species were not pooled together. Immature stages that did not yield adults by the fourth day were pooled based on location (maximum 25 larvae per tube).

#### Adult Mosquito Collection Methods

Two adult mosquito sampling methods were conducted simultaneously in all study sites, i.e., morning resting and human landing collection, to which a third one, an animal-baited trap, was added in one location (Malang, East Java).

(1) Morning resting collections were made by eight collectors using hand nets and aspirators. Collections were conducted from 7 to 9 a.m. and included any resting locations within the house. All adult mosquitoes were placed into labeled paper caps and taken to the field laboratory for further analysis.
(2) Human landing collections were performed by eight local volunteers as collectors in three selected houses in each study site for sampling adult mosquitoes using mouth aspirators. They were all trained before collecting mosquitoes. Three teams of two people sampled outdoors (up to 5 m from the house) and indoors. Each collector sat on a chair while exposing the legs. Sampling was conducted all night from 6 p.m. to 6 a.m. The teams rotated and changed roles regularly every 2 h with a 2-h break. Although the targeted *Aedes* mosquitoes are diurnal, Indonesia law does not allow human landing collection during daytime. Therefore, collections had to be conducted at night. This introduces a strong bias in the sampling, but because it is what surveillance teams do in accordance with the law, this method was nevertheless considered. Mosquitoes that have been collected per hour were then taken to the field laboratory for species identification and further analysis.
(3) An animal-baited trap was conducted by using tame animals, i.e., cows, placed inside a net all night. Mosquito collections were carried out for 15 min/h inside the nets by three collectors. Collected mosquitoes were then similarly preserved as for the human landing collection method.

All mosquitoes from these three collecting methods identified as *Ae. aegypti* and *Ae. albopictus* were then killed with ethyl acetate, pooled up to 25 mosquitoes in labeled 1.5-ml vial tubes with RNAlater (Qiagen, Hilden, Germany) and preserved based on the same cold chain management as above for larvae.

### Detection of Dengue Virus From Mosquitoes

The *Ae. aegypti* and *Ae. albopictus* mosquito pools were homogenized in 1.5-ml tubes containing 200 μl PBS 1x by using pellet pestles. RNA was extracted using QIAamp®Viral RNA Mini Kit (Qiagen®, Courtaboeuf, France). RNA was extracted from 200-μl homogenized samples following the manufacturer's instructions. All RNA extracted samples were analyzed for dengue detection using Lanciotti's protocol (23). The nested RT-PCR for dengue was performed using SimpliAmp Thermal Cycler Applied Biosystems™ (ThermoFisher Scientific®, United States). Amplification of dengue RNA was carried out with following specific primers: D1 (5′-TCA ATA TGC TGA AAC GCG CGA GAA ACC G-3′), D2 (5′-TTG CAC CAA CAG TCA ATG TCT TCA GGT TC-3′), TS1 (5′-CGT CTC AGT GAT CCG GGG G-3′), TS2 (5′-CGC CAC AAG GGC CAT GAA CAG-3′), TS3 (5′-TAA CAT CAT CAT GAG ACA GAG C- 3′), and TS4 (5′-CTC TGT TGT CTT AAA CAA GAG A-3′). The first amplification of dengue virus was performed using Superscript III one-step RT-PCR kit (Invitrogen, Carlsbad, CA). The cycling conditions consisted of an initial 95°C denaturation step for 2 min, followed by 40 cycles of 95°C denaturation for 30 s, 60°C annealing for 1 min, and 72°C extension for 1 min 30 s, and a final extension step 72°C for 10 min. Samples were then stored at 4°C. First-step PCR products were run on 2% agarose gel under 120 V current for 1 h. Visualization was done using SYBR® safe DNA gel stain (Invitrogen, Carlsbad, CA, USA) under UV condition in a GelDoc system. The presence of the 511-bp control band indicated a dengue virus (DENV) positive sample. Subsequent genotyping was conducted by using the first step PCR product with thermal cycle setting as follow: initial denaturation step at 95°C for 2 min, followed by 10 cycles of denaturation step at 95°C for 30 s, 60°C annealing for 1 min, and an extension step at 72°C for 1 min and 30 s. The final extension step was conducted at 72°C for 10 min. Subsequently, samples were stored at 4°C. Multiplex genotyping reactions yielded a single specific band with the size of 482 bp for DENV-1, 119 bp for DENV-2, 290 bp for DENV-3, and 389 bp for DENV-4. All field samples were tested for the presence of dengue virus after being pooled by 25 individuals of the same species.

### Dengue Incidence Data

The incidence, number of new dengue cases per total population for the time of the study, was obtained from the district health center in each district.

### Statistical Analyses

A first factor analysis of mixed data (FAMD) (24) was conducted using the incidence data, the number of mosquitoes, and the number of positive pools for each dengue serotype as quantitative parameters and mosquito species, methods of collection, and provinces as qualitative parameters. The effectiveness of the collection methods (qualitative data) against mosquito species (quantitative data) was assessed using a second FAMD. These analyses were performed using the R software with FactoMineR (25).

## Results

### Mosquito Sampling

A total of 44,675 mosquitoes were collected from 39 locations (Figure 1, Supplementary Table 1). Out of these 44,675 mosquitoes collected, 32,525 (72.8%) were *Ae. aegypti* and 10,300 (23.1%) were *Ae. albopictus*, and 1,850 (4.1%) were undetermined. When considering the method of capture, the highest number of captured individuals was, as expected, obtained when targeting larvae. The single larva method was the most efficient in terms of number of individuals collected. A total of 36,500 larvae were collected with this method out of which 27,475 were *Ae. aegypti*, 7,775 were *Ae. albopictus* and 1,250 were not identified. Out of 6,450 larvae collected and reared, 4,325 were *Ae. aegypti*, 1,575 were *Ae. albopictus*, and 550 were not identified. With both larval methods, a bias was observed in favor of *Ae. aegypti*, which represented 75.27% and 37.05% of all samples for the single lava and rearing methods, respectively. Very different results were obtained with the adult capture method. From the three methods used, human landing was the most efficient even though a bias was introduced by the legal obligation to perform this approach by night. Out of 1,325 adult mosquitoes captured, 325 were *Ae. aegypti*, 975 were *Ae. albopictus* and 25 were not identified. The animal-baited trap method yielded only 25 mosquitoes, all being *Ae. albopictus*. The ratio between *Ae. aegypti* and *Ae. albopictus* was reversed with this time a bias in favor of *Ae. albopictus*. It represented 73.58% and 53.33% for the human landing and morning resting methods, respectively. The animal baited trap method yielded only *Ae. albopictus*, but considering the very low number of mosquitoes captured, i.e., 25, this is not significant.

### Distribution of Dengue Virus

A total of 89 pools were positive for dengue virus. The most frequently encountered virus was DENV2 (*n* = 44), which made up half of the positive samples. DENV3 and DENV1 followed with 20 and 17 positive pools, respectively. DENV4 was detected in only one pool. Combinations were also detected. Eight pools contained a combination of DENV1 and DENV2, whereas the combination of DENV1 and DENV3 was found in only one pool. Another single pool contained the triple combination DENV1–DENV2–DENV3. With respect to the geographic distribution, a strong imbalance was observed. A large part of the detected dengue viruses, i.e., 56 (63%), were found in mosquitoes collected in the province of Aceh. All four dengue virus serotypes and all positive combinations were found in this province. The other provinces where positive pools were detected were West Sumatra (*n* = 5), Lampung (*n* = 6), Bangka-Belitung (*n* = 4), West Kalimantan (*n* = 2), South Kalimantan (*n* = 1), North Sulawesi (*n* = 2), East Java (*n* = 7), and Maluku (*n* = 6). A strong imbalance was also observed when considering the nature of the positive samples. Mosquito larvae were almost the exclusive source of virus, i.e., 93.3% (*n* = 83) with 70.8% (*n* = 63) found with the single larva method and 22.5% (*n* = 20) for the rearing method. Only six pools (6.7%) of adult mosquitoes were found positive with the human landing method totalizing two pools (2.3%), and four pools (4.4%) were found positive in mosquitoes collected with the morning resting method. An imbalanced result was also found regarding the mosquito species with 76.4% (*n* = 68) of the positive pools corresponding to *Ae. aegypti* and 23.6% (*n* = 21) corresponding to *Ae. albopictus*.

### Correlation Assessment

**Figure 2:**
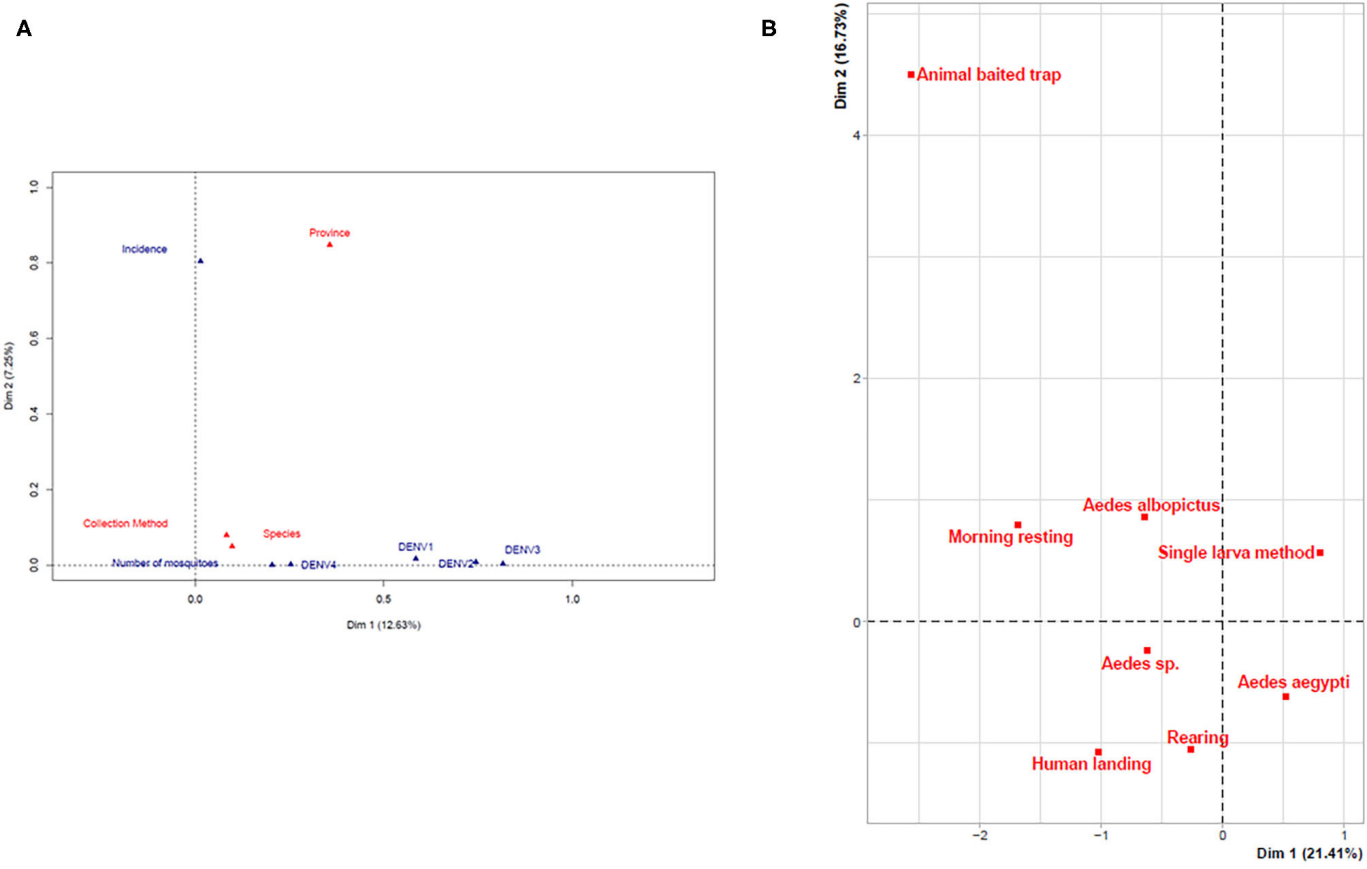
Multivariate analysis of parameters. **(A)** Global factor analysis of mixed data. **(B)** FAMD assessment of the effectiveness of the collection methods.

A FAMD was performed to determine the potential correlation between the various parameters considered: mosquito species, province, number of mosquitoes, collection method, dengue virus, and dengue incidence (Figure 2A). The only correlation that could be found was between the province and the incidence (Figure 2A). However, the global level of explanation was low (20%) indicating a lack of correlation between any of the parameters with the exception of province and incidence of dengue. A similar result was found when comparing the different collection methods with the mosquito species. The only, but rather weak, correlation that could be found was the preferred association of the larval methods with *Ae. aegypti* and the adult methods with *Ae. albopictus* (Figure 2B, Supplementary Table 1).

## Discussion

In the absence of commercialized vaccines and of any medical treatment, the management of dengue relies only on mosquito control and on prevention. Finding efficient and reliable descriptors for assessing the risk of dengue outbreaks is, thus, a priority in all dengue-endemic countries. The main tools currently in use for assessing this risk of dengue outbreak are the *Stegomyia* indices (15, 26), which rely on the calculation of the relative density of mosquito larvae present in containers and in households through the CI, HI, and BI (26, 27). However, these indices were shown to display no correlation with dengue infection rates and are, thus, not reliable descriptors (16, 18, 28, 29).

Because the *Stegomyia* indices are not reliable descriptors, they must be replaced by other descriptors. They could be replaced by other entomological indices provided that these other entomological indices are reliable. Entomological indices are, by definition, based on the capture of insects. Therefore, it is essential to assess whether the collection methods are reliable and reproducible and do not generate biases. This is independent from the calculation model applied. It is an intrinsic trait of the collection method itself. If not, entomological indices cannot be used as predictors. They must also be compatible with the logistical and administrative constraint of the “one point/one day” nature of the operational monitoring and decision process. This operational constraint is essential. Agents conducting mosquito surveys do not spend all their time at the same place and make their calculations and assessments and release their conclusions usually within 1 day. They move to another place as soon as the threshold of 100 households needed for calculation of the official *Stegomyia* indices is reached. The window for deciding on treatment is also narrow because treatments must be effective before outbreaks occur. Long-term assessments within a single place are scientific experimentations for the purpose of understanding biological processes or developing techniques and methodology but are not adapted to fast decision making. They are not suitable for use under such conditions. This constraint is even more important in large countries such as Indonesia. As a consequence, trapping methods, which are long-term methods based on cumulated data, are not favored.

The methods implemented being used to generate descriptors must be reliable regardless of the location and local conditions. We, therefore, conducted this large-scale study to assess the procedures endorsed by the Ministry of Health guidelines within a very large study involving many sampling sites corresponding to different urbanistic and geographical conditions. This work is, to our knowledge, the most extensive one with 44,675 mosquitoes collected in 39 different sampling locations over Indonesia within a short 2-month period, allowing thus a robust statistical analysis. The main conclusion of this work is that it is not only the *Stegomyia* indices, but any kind of entomological indices that might be at best of very limited use. Not only could no correlation be found between any methods and the presence of dengue virus in mosquitoes, but no correlation could be found between any of the methods and the incidence of dengue. The higher proportion of *Ae. albopictus* found in the human landing method might be related to the fact that this species is more crepuscular than *Ae. aegypti*. No consistency was found for any given method from one place to another. Finally, there was no consistency in the efficiency of a given method for detecting dengue. The single-larva and rearing methods yielded 63% of all dengue-positive samples in the sole province of Aceh. However, the incidence of dengue in Aceh is not the highest among all provinces and is rather the average. Provinces displaying the highest incidence, such as Bangka-Belitung, South Kalimantan, or North Sulawesi, did not yield any dengue-positive larvae. The only single positive pool in these provinces was found in South Kalimantan among morning resting adults. This lack of correlation between incidence and dengue infection rate in mosquitoes is also a drawback for methods associating the capture of adults and the direct detection of dengue virus in the sampled mosquitoes (30–32).

The use of *Stegomyia* indices and the monitoring of mosquitoes are today the main means of assessment of the risk of dengue outbreaks and efficiency of mosquito control. Previous studies demonstrated the lack of correlation of the *Stegomyia* indices with the risk of dengue outbreaks and dengue incidence (12, 14–18). In this work, we further demonstrate the lack of consistency of the various collection methods officially recommended and the lack of correlation with the recorded dengue incidence. Altogether, this indicates that entomological approaches do not provide reliable indicators of risks of dengue outbreak. The risk with these methods is mostly misleading interpretation and misguided decisions and allocation of resources. The assessment of the risk of dengue outbreaks should be reconsidered from a different angle.

## Conclusions

A previous study found that a factor positively correlated with the incidence of dengue was the human population density (18). This provides an angle for further research. *Ae. aegypti* and *Ae. albopictus* are both anthropophilic species, and the human environment is, thus, a major driver of their demography and distribution. The measurement and prediction of the risk of dengue outbreaks should be considered from the angle of urbanism and human societal factors. Efforts should be devoted to the development of novel societal indices. It is even more important to communicate on this issue because dengue endemic countries worldwide, as well as WHO, still base their recommendations and dengue management procedures on entomological indices.

## Data Availability Statement

The datasets presented in this study can be found in online repositories. The names of the repository/repositories and accession number(s) can be found in the article/Supplementary Material.

## Ethics Statement

The studies involving human participants were reviewed and approved by the Ethical Commission Board of the NIHRD, Ministry of Health, Indonesia (No. LB.02.01/5.2/KE.003/2016 and No. LB.02.01/5.2/KE.020/2017). The patients/participants provided their written informed consent to participate in this study.

## Author Contributions

TG, LS, TS, RW, and MP conceived and designed the field studies. MujiyonoM, SN, and DS prepared samples. TG and MP ran molecular analyses and laboratory experiments. TG, LG, and RF analyzed the data. MujiyantoM prepared the map. TG and RF wrote the manuscript. SM, LG, and RF provided critiques and significant revisions to the manuscript. All authors contributed to the article and approved the submitted version.

## Conflict of Interest

The authors declare that the research was conducted in the absence of any commercial or financial relationships that could be construed as a potential conflict of interest.

## Supplementary Material

Supplementary Table 1 Sampling localities and characteristics of samples.

## Abbreviations

HI: House Index
CI: Container Index
BI: Breteau Index
FAMD: Factor Analysis of Mixed Data.
